# Digital entrepreneurial intentions and actions in China during the COVID-19 pandemic with policy implications

**DOI:** 10.1177/03063070231172267

**Published:** 2023-04-19

**Authors:** Zhi-xing Xu, Ying Zhu, Jingting Liu, S Tamer Cavusgil

**Affiliations:** Business School, 546205 Beijing Normal University, Beijing, China; Australian Centre for Asian Business, 64771 The University of South Australia, Adelaide, AU-SA, Australia; CU Denver Business School, 33882 University of Colorado-Denver, Denver, CO, USA; J Mack Robinson College of Business, 1373 Georgia State University, Atlanta, GA, USA; Leeds University Business School, Leeds, UK

**Keywords:** China, contextual environment, COVID-19, digital start-ups, entrepreneurial intentions and actions, policy implications

## Abstract

Despite the rapid development of digital start-ups and market expansion in China, there have been challenges for developing digital business in recent years. Unique economic, institutional, and social factors, as well as the recent COVID-19 pandemic, influence the digital entrepreneurs and their businesses. Yet, the literature on the changing digital entrepreneurial behaviour during the COVID-19 pandemic remains limited. The intentions and behaviours of these entrepreneurs in relation to their digital start-ups and the impact caused by exogenous changes require deeper investigation. By adopting an intention-based social cognitive perspective, this study examines the factors influencing digital entrepreneurs’ intentions and actions in managing their start-ups. We also present a holistic framework with regard to the changing entrepreneurial behaviour and policy implications for the development of digital start-ups.

## Introduction

As Pidduck and Tacker (2022) point out, advancing knowledge on the critical inputs to entrepreneurship requires entrepreneurship scholars to explicitly seek out and probe the so-called meaningful heterodoxies. These heterodoxies refer to the socio-cultural settings and/or potentially contentious phenomena encountered by entrepreneurs that can be influential for generating novel and valuable ways of solving problems. The COVID-19 global pandemic which started at the beginning of 2020 has shaped the new reality of the business world. The magnitude and scale of the COVID-19 pandemic have tremendously changed the way businesses act and consumers behave (Wenzel et al., 2020). The reasons for these changes include long-term home stay, isolation measures, and political, economic, and social policy uncertainty. This crisis is bound to have a wide and far-reaching social and economic impact which may fundamentally change the way firms do business (Donthu and Gustafsson, 2020).

In the area of the digital economy, consumers and the entire society have expected to see socially responsible digital firms offer reliable and high-quality products and services during the pandemic (Song et al., 2021; Song, 2022). Designing a new business model has become an important tool to improve business survivability and ecological adaptability to cope with ever-changing societal norms, technology, and product market environments (Amit and Zott, 2015, 2020). New opportunities may also be found among the challenges. The ‘new reality’ during the COVID-19 pandemic urges entrepreneurs and business leaders to rethink and systematically re-design business models which are more inclined to resilience rather than efficiency through the adoption of new digital technology (Ratten, 2020). In other words, these changes may stimulate business model innovation and increase the need for entrepreneurs to formulate business models based on innovation strategies (Amit and Zott, 2020).

The literature on the characteristics of digital entrepreneurship during the COVID-19 pandemic remains limited (Garcia et al., 2021). So far, scholarly work on digital entrepreneurship frameworks has focused on certain elements of the external factors, such as the regulatory climate and business ecosystems, and the internal factors – namely, entrepreneurial traits in relation to adopting venture opportunities and strategies (Shi et al., 2022; Zaheer et al., 2019). However, the adoption of a particular digital business model is more complex due to the interplay among multiple elements. These elements include individual entrepreneurs’ characteristics and choices in responding to the contextual factors, such as stakeholder activities and environmental constrains (Amit and Zott, 2015; Andries et al., 2013). Entrepreneurs’ inner goals to create and capture value, and the external incumbents and environmental constrains are two drivers of business model design (Amit and Zott, 2015; Amit and Han, 2017).

Scholars have called for research focusing on the interplay between these important aspects (Gorgievski and Stephan, 2016; Su, 2021; Zhu et al., 2021) as well as the potential implications for policy and/or regulations (Massaro et al., 2016; Zaheer et al., 2019). Thus, we complement the current literature regarding the determining factors affecting digital entrepreneurs’ thinking and behaviour in the process of launching digital start-ups during the COVID-19 pandemic (Bradley et al., 2021). In order to address the key factors regarding the interplay between entrepreneurial intentions/actions and typical exogenous influences during the COVID-19 period, the present inquiry adopts the intention-based social cognitive perspective. Intention-based approaches have been central to entrepreneurial intention (EI) research to date; thus, we introduce a new conceptual framework based on a typical exogenous context (Esfandiar et al., 2019).

By investigating digital entrepreneurs operating start-ups during the COVID-19 period, we present a holistic framework for studying entrepreneurial intentions and actions, and inter-related factors influencing digital start-ups in China. We aim to address the following fundamental question: how do digital entrepreneurs interact with changing contextual environments in conceiving and launching digital start-ups during the COVID-19 period?

In order to shed light on the development of the digital entrepreneurship phenomenon, we carried out fieldwork interviews with 10 digital start-ups in 2021. We followed their business operations, observing their decision-making through business meetings, semi-structured interviews with the business owners as well as collecting secondary data – company brochures, reports, and archival documents. We also complemented this research with in-depth conversations with industry and academic observers of this contemporary phenomenon. Finally, we benefited from the extant literature, including business press coverage of digital entrepreneurship.

## The literature

This study is designed to respond to the recent call for new studies that conceptualize the interplay of internal and external factors as meaningful heterodoxies within a unique socio-cultural setting (i.e. China) and investigate the contentious phenomena (i.e. the influence of COVID-19 on changes in social norms and policies) encountered by entrepreneurs (Pidduck and Tacker, 2022). We link the elements of personal intention and subjective norms with perceived behavioural control. This approach enables us to address inner and outer challenges faced by individual entrepreneurs, and opportunities in the process of developing digital start-ups and interacting with the unique contexts. The present review of literature defining digital entrepreneurship and the interplay between inner and outer factors determining the survivability of digital start-ups has taken the influence of the global pandemic into account.

### Digital entrepreneurship and the interplay between inner and outer factors

Digital entrepreneurship differs from general entrepreneurship and can be defined as ‘the process of entrepreneurial creation of digital value through the use of various socio-technical digital enablers to support effective acquisition, processing, distribution, and consumption of digital information’ (Sahut et al., 2021: 4). This definition can be extended and applied to different types of ventures as well as used to support different processes of new venture creation. New technologies enable digital entrepreneurs to reduce the barriers between invention and creation of a new enterprise (Steininger, 2019). Hence, according to Le Dinh et al. (2018: 1), digital entrepreneurship is ‘the reconciliation of traditional entrepreneurship with the new way of creating and doing business in the digital era’.

As Nambisan et al. (2017) point out, the digital eco-system, including regulatory policies, digital infrastructure, venture capital, and competitive and innovative environments, can provide numerous promising opportunities for entrepreneurs, but it could also constrain their development. Several contingencies have been identified with impact on shaping the development of digital entrepreneurship; these include digitization (the relevant digital technologies of artefacts, platforms, and infrastructure), a broader set of institutional arrangements (innovation intermediaries, crowdsourcing/funding, and makerspaces), individual entrepreneurial capabilities/competencies, regulations and policy-related factors, and globalization and entrepreneurship (research on digital platforms in Western, primarily U.S., vs. Chinese settings) (Nambisan et al., 2017).

Another important area of digital entrepreneurship study has been the digital business model (DBM) (Amit and Zott, 2015; Sahut et al., 2021). This research stream focuses on the differences between DBM and a general business model, with a description of new DBM typologies, enabled by digitalization and the associated challenges and opportunities inherent in the emergence of DBMs (Margiono et al., 2018; Richter et al., 2017). Certainly, the adoption of a particular DBM can also be the outcome of individual entrepreneurs’ characteristics and choices in responding to the contextual factors, such as the preference of VC investors or regulatory environments (Amit and Zott, 2015; Andries et al., 2013). Once again, the importance of the interplay between some inner and outer factors influencing digital start-ups is demonstrated, albeit not in a comprehensive manner. Therefore, it is our intention to develop this research to complement the current literature regarding the determining factors affecting digital entrepreneurs’ thinking and behaviour in developing digital start-ups during the COVID-19 pandemic with the influence of policy shifts and social/economic circumstances (Bradley et al., 2021). The underpinning of the intention-based social cognitive perspective provides the link and elaboration regarding these key elements in our research.

### Intention-based social cognition literature

The questions regarding the reasons for an individual choosing to engage in an entrepreneurial career in general, and the young generation venturing into digital start-ups in particular, are cognitive in nature. According to the literature on social cognition, career choice is best predicted by intentions (Lent et al., 1994), and an entrepreneurial career, in particular, can be described as an entrepreneur’s intentions to form a new venture instead of joining an existing one (Davidsson, 1991; Katz, 1992; Krueger et al., 2000). In order to understand these choices, we have to explore intention and antecedents. The issue of antecedents of entrepreneurial intention (EI) has been addressed by different scholars from both an individual and an environmental perspective. Approaches have included the influence of personality and psychological factors on EI as well as the contextual influence of policy, industry norms, and overall business eco-system (Bacq et al., 2017; Liñán and Fayolle, 2015; Zhao et al., 2010), and possibly sudden events such as wars or pandemics (Garcia et al., 2021).

Prior entrepreneurial research has attempted to identify the social, cultural, political, and economic contextual factors that encourage new venture development, such as job displacement (Shapero and Sokol, 1982), previous work experience (Mokry, 1988), quality of urban life (Pennings, 1982), and ethnic group membership (Greenfield and Strickon, 1981). Bruno and Tyebjee (1982) identify several contextual factors that may stimulate entrepreneurship, including the availability of VC, governmental influences, accessibility of customers, suppliers and transportation, and the availability of resources such as a skilled labour force, land and facilities, and other support services.

In terms of intention-based research, many scholars have focused on identifying the individual predictors of intentions to engage in entrepreneurial activity according to the theory of planned behaviour (TPB) (Ajzen, 1988, 1991; Ajzen et al., 2009). A central factor in the TPB is the individual intention to adopt a given behaviour. According to Ajzen (1991: 181), intentions are assumed to capture the motivational factors that influence a behaviour. The TPB has been frequently applied in the study of EI to explain the antecedents that shape individual intention to pursue entrepreneurship (Díaz-García and Jiménez-Moreno, 2010; Liñán, 2008; Liñán and Chen, 2009; Moriano et al., 2011).

As Shinnar et al. (2018) point out, in one-third to nearly half of the variations in EI recorded in various studies, the three cognitive antecedents of the TPB were considered to be reliable predictors of EI. However, EI and its antecedents are only important if they lead to meaningful outcomes, and in the field of entrepreneurship, that could be running start-ups with different behaviours. However, the intention-behaviour based approach has a number of limitations, such as only focusing on intention to engage in entrepreneurial activities with short time intervals (Shinnar et al., 2018). In addition, Bacq et al. (2017) point out that this approach does not account for the role of exogenous influences on intentions or behaviours. Hence, these researchers call for further study to be developed of such interactions between cognitive, behavioural, and environmental factors.

In contrast, Shapero’s entrepreneurial event (SEE) approach (Shapero and Sokol, 1982) posits that the intention to initiate an entrepreneurial event such as establishing a new venture requires three critical antecedents: (1) perceptions of desirability; (2) perceptions of feasibility; and (3) propensity to act (Shapero and Sokol, 1982). Personal and social EI-related feasibility and desirability themselves are influenced directly by self-efficacy and personal desirability, respectively. Desirability and feasibility are to some extent conceptually homologous to attitude and perceived behavioural control in the TPB, respectively. The addition of propensity to act derived from the SEE leads to capturing the potential for identification and recognition of a credible new venture opportunity in the markets. Acting on credible entrepreneurial opportunities is vital given that favourable attitudes and social norms towards an EI are not sufficient to become intent to activate an entrepreneurial event. According to Shapero and Sokol (1982), understanding the entrepreneurial process requires an understanding of how credible opportunities are created in the markets. Hence, based on the SEE approach, a credible entrepreneurial opportunity hinges on two important antecedents − perceived desirability and perceived feasibility with a certain degree of propensity to act on these opportunities. In terms of the exogenous influences on EI, later research, such as Krueger et al. (2000), demonstrated that the exogenous variables of the TPB do not explain entrepreneurial intention as strongly as the exogenous variables in SEE. Hence, it is expected that new research on developing new frameworks may be valuable based on the combination of social norms and self-efficacy derived from the TPB, and the propensity to act with desirability and feasibility from the SEE approach (e.g. Esfandiar et al., 2019).

### The pandemic’s influence on policy and digital start-ups

Recently, further studies on the impact of the COVID-19 pandemic on policy and economy in general, and entrepreneurship in particular, have been published, including a number of special issues (Ashraf et al., 2022; Belitski et al., 2022; Grassi and Fantaccini, 2022; Narayan, 2022; Taylan et al., 2022). These recent publications shed light on the economic effects of the pandemic by looking at the macro- and micro-economic effects on economic policy and the survivability of entrepreneurial start-ups as well as the role of financial support, public funding for well-being in both developed and developing countries.

The key aspects being addressed include the macro-economic effects of COVID-19 on the way of living and working, as in Zhang et al. (2022) and Meurer et al. (2022), who demonstrate how small businesses and individual entrepreneurs can adjust to new business conditions by working from home, developing new business models, and seeking social support to leverage the negative impact of the COVID-19 pandemic. Another study by Pedauga et al. (2022) takes a macro-economic perspective to empirically test the role of small business in the economy. A second important area of study is the economic and non-economic impact of the COVID-19 pandemic on small business performance, as in Grözinger et al. (2022) and Torrès et al. (2022) who demonstrate the increased risk of burnout during the pandemic and that financial threat is the dominant cause. Kalenkoski and Pabilonia (2022) examine the initial impacts of COVID-19 on employment and hours of unincorporated self-employed workers. Richter and Patel (2022) complement prior research on the self-employed from the perspective of racial minority groups and extend the argument that minorities may face greater adversity from the COVID-19 pandemic in developed countries.

A third important area of study is the role of financing for entrepreneurships during crises, and a variety of support tools (Dushnitsky et al., 2020). Liu et al. (2022) examine Chinese SMEs’ financing responses to the outbreak of COVID-19. Fairlie and Fossen (2022) investigate the effect of the US federal government’s response to assist small businesses, and Atkins et al. (2022) provide further understanding of the role of race in loans made through the Paycheck Protection Program (PPP). Block et al. (2022) investigate the measures that entrepreneurial ventures take to preserve liquidity, and Dörr et al. (2022) focus on fiscal policy in rescuing companies short of liquidity due to insolvency.

The fourth stream of study represents a variety of micro and macro public support and well-being programs aimed at mitigating the negative effects of the COVID-19 crisis, including a study by Lastauskas (2022) which examines businesses’ employment adjustments after the imposition of stringent lockdowns in March 2020. Belghitar et al. (2022) examine the impact of COVID-19 on UK small businesses and how government intervention affected their ability to survive the pandemic, and a study by Braunerhjelm (2022) deals with macro-economic stabilization policies. Tanner et al. (2022) evaluate the internal and external factors that enable SMEs to (re)build resilience during the COVID-19.

### The background of policy shift and impact on digital start-ups in China

The spread of COVID-19 has pushed the ‘pause button’ of economic development in China. Due to the rapid spread of the pandemic and its widespread destructiveness with considerable impact on people’ life, the Chinese government has launched an emergency response from the top to bottom, adopting a series of emergency response measures to prevent and control the pandemic. These measures have had a very negative impact on the economy. According to the data released by the National Bureau of Statistics of China, the GDP in the first quarter of 2020 fell by 6.8% year-on-year, the national service industry production index fell by 13.0% year-on-year, the total retail sales of consumer goods fell by 20.5% year-on-year, and the national fixed asset investment fell by 24.5% year-on-year. Due to the stringent lockdown policy, many companies were confronted with issues like supply chain disruption, increased labour costs, higher foreign trade risks, and drying up of cash flow. A large volume of offline consumption was suppressed, having a great impact on SMEs in catering, accommodation, leisure, and other industries that rely on offline consumption. The inherent vulnerability of SMEs leads to them having low resilience in this crisis. The zero-COVID policy, and massive intensive lockdowns and restrictions removed any expectations of a stable future, and entrepreneurs lost control of their enterprise operations. It is well established that entrepreneurship is hindered by increased uncertainty, a rise in action-related fear and doubt (Gelderen et al., 2015). Fewer people are willing to start businesses and expand reinvestment during the pandemic era in China.

At the same time, the outbreak of the pandemic has increased online business and online marketing opportunities. Online platforms have improved the ability of enterprises to work in remote collaboration. Small- and medium-sized enterprises have actively embraced digital technology and digital transformation, and improved their digital capabilities. Many large enterprise platforms, such as Alibaba and Tencent, have also opened or provided mature digital platforms and technologies to help small- and medium-sized enterprises cope with the negative impact of the pandemic. Many SMEs chose to use online office platforms for business collaboration, video conferences, and work scheduling. In addition, the global spread of the pandemic has further changed consumption demand and consumption mode. Personalized, customized, and diversified demand has become increasingly common, and the far-reaching effect of consumption increasingly obvious. Digital transformation became an important way to promote the innovative development of SEMs, provide the tools for refined management, and decision-making. The outbreak of the pandemic has promoted the continuous progress and innovation of digital information technology; it has accelerated the transformation of business models and has become critical for the industry to accelerate digital transformation.

To date, there has been an increase in the literature on the interplay between exogenous factors and impact on entrepreneurial activities during the COVID-19 period. However, there is a need for further research to develop a better understanding by comparing these factors, in particular in the areas of policy shifts, challenges, and opportunities associated with the sudden event of the pandemic. Therefore, we develop the present study to examine the factors that influence digital entrepreneurs’ intentions/actions through interaction with exogenous factors in the process of developing digital start-ups in the crucial period of COVID-19. By doing so, we can delineate the intentions and actions of digital entrepreneurs’ interplay with the changing societal contexts.

## Research design

### Research plan and data collection

Given the underspecified nature of the phenomenon being studied and in addition to examining contextual implications, we carried out in-depth qualitative case studies to explore the relevant issues as well as develop alternative interpretations of the digital entrepreneurship phenomenon (Hesse-Biber and Leavy, 2011; Miles and Huberman, 2004; Yin, 2008).

Our research plan included a fieldwork plan consisting of case selection, observation, an interview protocol, and secondary data collection. Since samples in qualitative research tend to be small in order to support the depth of case-oriented analysis that is fundamental to this mode of inquiry (Sandelowski, 1996), we believe a selection of ten digital start-ups as case studies is sufficient to collect richly textured information, relevant to the phenomenon under investigation as purposive sampling, rather than probability sampling employed in quantitative research (Patton, 1990). We used our university start-up networks to select ten digital start-up firms in different locations with different sizes, business duration, and products/services. The nature of the ten digital start-up business operations includes digital media and marketing, online digital education and training, digital movies and games, data processing, and online finance. The detailed profiles are presented in Table 1.

**Table 1.** Profiles of digital start-ups.

ID	Age	Business	Size (no. of employees)	Owners’ experience	Operation details
C1	1	Digital media	2	International education consultant for 2 years	A start-up firm on digital media design and online promotion of English study materials with publishing houses
				Manager at a private education agent for 7 years	
				Developing start-up business for 1 year	
C2	8	Digital education	15	Research assistant at a pre-school children’s education institute for 2 years	A start-up firm offering online education and developing videos for online causes
				Researcher and trainer at a private education company for 2 years	
				Developing start-up businesses for 8 years	
C3	3	Digital marketing	11	Management in online shopping APP development for 9 years	A digital start-up providing e-commerce/marketing and developing online customers for large online platforms
				Management in product development at a private firm for 5 years	
				Developing start-up business for 3 years	
C4	1	Digital TV program development	4	Brand and marketing management at China Central TV Net for 10 years	A digital start-up developing new online TV program
				CEO of a TV program production for 4 years	
				Developing a new start-up business for 1 year	
C5	4	Digital advertising	6	Editing and customer management at China Commerce Net for 3 years	A digital start-up providing online advertising design and promotion for large companies
				Sales manager at a consumer guide newspaper for 7 years	
				Sales manager at China Sohu for 2 years	
				Developing a new start-up for 4 years	
C6	3	Digital health services	21	Providing rehabilitation plan for patients at a health clinic in Ningbo city for 3 years	A digital start-up focusing on online health care services and providing rehabilitation management packages to large online platforms
				Developing a start-up for digital health care services for 3 years	
C7	7	Digital trading	7	Manager at a tea company shop for 4 years	A digital start-up firm focusing on trading tea online
				Working as an e-commerce director at a trade company for 7 years	
C8	3	Digital international trade	8	Working as a marketing manager at an education and technology company for 3 years	A digital start-up firm providing cross-country e-commerce with Russia
				Developing a digital start-up on cross-country trade for 3 years	
C9	7	Digital finance	35	Developing an international education agent in 2010	A digital start-up firm providing financial support and online payment
				Developing a start-up for digital payments for international students for 7 years	
C10	10	Digital coaching for new businesses	90	Managing an online gaming platform for 3 years (2009–2011)	A digital start-up firm providing online platforms to support the development of new start-ups
				Developing a VC platform supporting new start-ups in 2011	
				Developing a new digital platform for 10 years	

The authors started to visit these businesses in 2021, attending their business meetings as well as reading their company brochures, reports, and archival documents. Based on the understanding of their business operation, we developed semi-structured interview protocols and interviewed the owners of the ten firms. In addition, we discussed the relevant issues with industry and academic observers during the fieldwork. Hence, we were able to illustrate the crucial aspects of the research on the intentions and actions of digital entrepreneurs and how the entrepreneurs interact with the changing contextual environments to develop their digital start-ups.

The most crucial information was collected through in-depth semi-structured interviews with the owners of these ten digital start-ups. Each interview lasted about 1 hour; detailed interview records and transcripts were generated by the authors. In accordance with the interview guide, each interview started with questions about the background (i.e. individuals and their businesses) and motivation for developing a digital start-up business. Other issues addressed included the digital business and social norms, previous education and work experience influencing business operations, adoption of different business models, the role of external investors and their behaviour, government policy and business environment with related challenges and opportunities arising from the impact of COVID-19, and strategic responses.

### Analytical strategies

We employed a thematic approach to analyze the qualitative data (King, 2004). Codes were developed in relation to themes (Braun and Clarke, 2006), and analysis was performed in several steps as suggested by Cassell (2015) and Miles and Huberman (2004). By adopting cross-sectional analysis (Gioia et al., 2012), we analyzed the data with multiple iterations between the data and emerging theoretical themes. Based on Pratt et al. (2006), the data analysis comprised three main steps: classifying the raw data into first-order empirical codes, then consolidating the empirical codes into conceptual themes, and finally aggregating the conceptual themes into dimensions, namely, entrepreneurial ‘intentions and actions’ (Ajzen, 1991; Shapero and Sokol, 1982).

**Figure 1.**
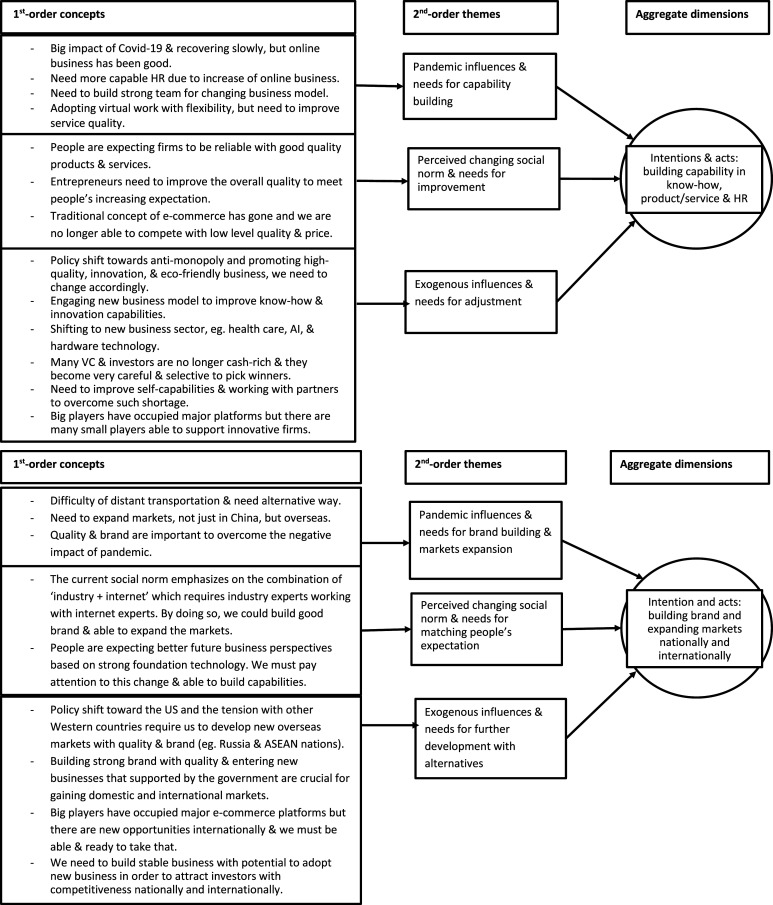
Entrepreneurs’ responses towards changing contextual environment under the pandemic.

In the first stage of data analysis, the authors selected and read the relevant statements from the interview transcripts, field notes, and secondary documents. At the same time, the authors started categorizing the raw data into first-order codes, using language that was as close to the data as possible. The authors then discussed the emerging themes and identity of the dimensions, namely, entrepreneurial ‘intentions and actions’. Following Pratt et al. (2006), we actively brainstormed alternative conceptualizations of how these dimensions related to each other and the literature before finalizing our model as shown in Figure 1, indicating entrepreneurs’ actions (both intention and action) to the contextual environments under COVID-19.

## Findings

### Entrepreneurs’ intentions and actions to the contextual environments under the COVID-19

With regard to the emerging contextual environments during the COVID-19 period, we paid particular attention to the changes brought by the pandemic, consequent policy shifts, the interplay between these changes, and entrepreneurs’ intentions and actions. In analyzing the findings, we compared the perspectives of the ten digital start-ups with regard to the impacts of the pandemic, perceived changing social norms, policies and investor behaviour, and the responses adopted by the start-ups. Figure 1 presents the statements made by these business owners, which helped us generate the relevant themes and dimensions, such as ‘intentions and actions on building capabilities, partnership and new business sectors/models’ and ‘intentions and actions on building brand and expanding markets’. The factors determining these intentions and actions are elaborated in detail in the following sections.

### Intentions and actions on building capabilities, partnerships, and new businesses

As Figure 1 demonstrates, according to the responses made by the ten start-up firms, the impact of the pandemic was mixed with negative effects and emerging new opportunities. For instance, in the business meeting, the owner of C2 claimed: ‘COVID-19 had a big impact, particularly in 2020. So far, the recovery has been slow, but the online business has been growing faster’. C3’s owner added that ‘the pandemic has promoted our online business, but the offline business has been slow. We need to shift our focus to improve our online products and services for further expansion’. Many others also made comments about making certain adjustments based on their previous educational or work experiences, as claimed by the owner of C5: ‘By using our previous knowledge and expertise, we could re-design our business by shifting 90 percent of business to online with better business results in 2021 than the results in 2019’. The owner of C10 added: ‘By adopting virtual work with more flexible management policy with our existing expertise, we could generate more efficient outcomes’.

With regard to the perceived changing social norms and need for improvement, a number of interviewees pointed out the changes in people’s expectations of the quality of products and services, and the need for further changes. For instance, C5’s owner claimed: ‘People are expecting firms to be reliable with good quality products and services’. C10’s owner added that ‘entrepreneurs need to improve the overall quality to meet people’s increasing expectations’. C8’s owner commented that ‘the traditional concept of e-commerce has gone and we are no longer able to compete with low level quality and price, but need to find alternative ways for business growth’.

In addition, policy shifts can be seen as the major exogenous influences in the post-COVID-19 period, as pointed out by C9’s owner: ‘Policy shifts towards anti-monopoly and promoting high-quality, innovation, and eco-friendly business, so we need to make changes accordingly’. C10’s owner also added that ‘new policy requires us to engage new business models to improve know-how and innovation capabilities’. C9’s owner also emphasized ‘shifting to new business sectors, such as health care, AI and hardware technology for achieving continuous growth’. People also commented on the changing attitude and behaviour of VC investors, as claimed by C4’s owner: ‘Many VC investors are no longer cash-rich and they become very careful and selective in picking winners’. C10’s owner also added: ‘The time of using PPT with presentations to get VC investment has gone. Investors are looking for a combination of real experience and future business perspective with lower investment risks’. In order to address these changes and challenges, a number of interviewees emphasized the ‘need to improve self-capabilities in the areas of know-how, quality of HR, products and services’ (C1), ‘working with partners to overcome such shortages’ (C8), and ‘big players have occupied major platforms, but there are many small players able to support innovative firms’ (C9). C10’s owner also emphasized the need for adopting new DBM: ‘Those who adopted an asset-light business model earlier are no longer able to develop into a sizable platform nowadays, but innovative firms could adopt different models with vertical integration with partners to explore new opportunities and achieve sizable platforms’.

Based on these comments, we can conclude that the changes in the post-COVID-19 period present mixed impacts with negative aspects and new opportunities. Consequently, digital entrepreneurs have shifted their intentions accordingly and have acted on building capabilities, partnerships in new business sectors and DBM, supported by policies for continuous growth.

### Intentions and actions on building brand and expanding markets

The shift of entrepreneurial intentions and actions on building brand and expanding markets (i.e. nationally and internationally) was also driven by the effects of the pandemic, perceived changing social norms, and other exogenous influences such as policy shifts and changing investor behaviour (see Figure 1). For instance, C8’s owner pointed out: ‘The pandemic influenced our international railway links with Russia and it has negatively impacted our cross-country trade’. Other people also emphasized ‘the need to expand markets, not just in China, but overseas’ (C9), and ‘developing high quality brand is important to overcome the negative impact of the pandemic’ (C5).

There were also changes in the perceived social norms and the need for matching people’s expectations. For instance, C10’s owner pointed out: ‘The current social norm emphasizes the combination of “industry + internet,” which requires industry experts working together with internet experts. By doing so, we could build a good brand and were able to expand the markets’. C9’s owner also emphasized that ‘people are expecting better future business perspectives in the markets based on strong foundation technology, and we must pay attention to such changes’.

Other exogenous influences were also discussed, such as the policy shift towards the US due to the geo-political tensions and trade war, and policy changes on supporting and not supporting certain business areas. For instance, C8’s owner explained that ‘policy shifts toward the US require us to develop new overseas markets with quality and brand (e.g. Russia)’. C9’s owner also added that ‘building a strong brand with quality and entering new businesses that are supported by the government are crucial for gaining domestic and international markets’.

With regard to the challenges presented by the major players, C10’s owner maintained: ‘Big players have occupied major e-commerce platforms, but there are new opportunities internationally and we must be able and ready to take advantage of those’. C1’s owner also added: ‘We need to build stable business with potential to adopt new business in order to attract investors with competitiveness nationally and internationally’.

Based on these comments, we can conclude that the changes in the post-COVID-19 period are challenging digital entrepreneurs’ conventional perceptions regarding the development of digital start-ups. Consequently, digital entrepreneurs have shifted their intentions and acted on building quality brands and expanding markets nationally and internationally by developing strong foundation technology, linking industry and internet expertise, and enhancing international collaboration for continuous growth.

## Discussion

**Figure 2.**
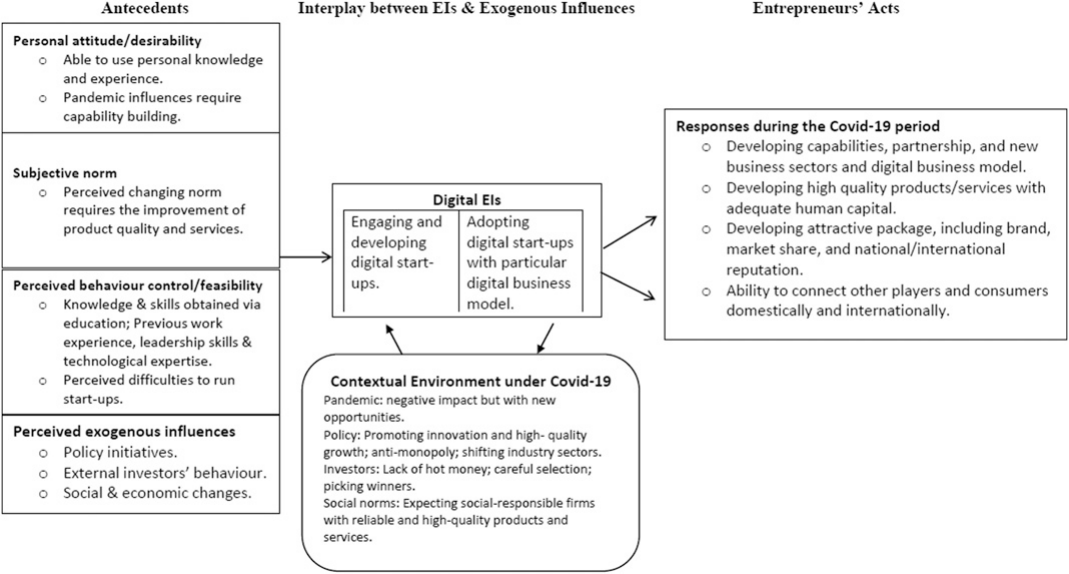
Developing digital entrepreneurship: Interplay between EI and exogenous influences.

Our research offers complementary perspectives on the factors influencing the digital entrepreneurial intentions and actions in developing digital start-ups during the COVID-19 pandemic. Our findings demonstrate major shifts in entrepreneurial behaviours through the interaction between cognitive and environmental factors. Based on the findings, we have developed a new framework for studying the development of digital entrepreneurship as it has played out during the COVID-19 period. This framework is depicted in Figure 2 and represents several implications for theory, policy, and practice which we elaborate in the following sections.

### Implications for theory

An understanding of the antecedence-based intention dimensions and exogenous influences on intentions and behaviour enables us to progress digital-entrepreneurship scholarship in multiple ways. Our research makes contributions to the recent call for developing new studies with a conceptual framework for the interplay of internal and external factors (Gorgievski and Stephan, 2016; Zaheer et al., 2019).

Our findings demonstrate that inner factors, such as personal attitude, subjective norms, and perceived behaviour control (i.e. self-efficacy), as well as external factors, such as policy initiatives and the behaviour of external investors, determine digital entrepreneurs’ objectives of engaging in and developing digital start-ups during the COVID-19 period. As Shinnar et al. (2018) point out, so far, only a handful of studies based on the TPB approach have examined the intention-behaviour link. These studies have a number of limitations, such as only focusing on intention to engage in entrepreneurial activities in short time intervals but not on other intention-entrepreneurial behaviours. Bacq et al. (2017) also point out that, despite the contributions of TPB in understanding the entrepreneurial process by offering a useful theoretical foundation to explain and predict intentions to create a new venture, TPB does not account for the role of exogenous influences on intentions or behaviours. Hence, the said researchers call for further studies to be developed on such interactions between cognitive, behavioural, and environmental factors. Examples of further investigations may include combining TPB with the SEE approach in a single model, by focusing on the propensity to act with desirability and feasibility from the SEE, and social norms and self-efficacy from the TPB (e.g. Esfandiar et al., 2019).

In this study, we combine TPB and SEE approaches and have been able to develop our findings beyond prior studies which were based on either the TPB approach or the SEE approach. Our study offers a holistic framework indicating the interplay of relevant entrepreneurial intentions and actions with exogenous factors in a particular social/cultural setting (i.e. China). The framework includes potentially contentious phenomena such as COVID-19 and the related changes of policy and business environments (Pidduck and Tacker, 2022). The findings demonstrate that digital entrepreneurs’ intentions and actions are not only based on assessing the possibilities internally but are also based on capabilities to evaluate emerging opportunities with the desire to expand business within the constrained exogenous environment. The propensity of digital entrepreneurs to act based on such interconnected elements determines the outcome of adopting a particular DBM and entering relevant business sectors (see the detailed illustration in Figure 2). These are good examples of a combined approach based on TPB and SEE.

However, the determination of multiple factors and links of antecedences and intentions with behaviours and outcomes requires investigation by future research from multiple perspectives and dimensions. Questions remaining open and needing to be answered in future research include the following: which of the observed characteristics are constant as an inherent trait defined by the digital industry and which are varying as the contextual conditions change; under what circumstances would these characteristics evolve and towards which other direction; should we expect different findings in cross-country comparative studies and with what theoretical implications?

### Implications for policy

Based on the research findings, it is evident that there are many challenging issues facing policy makers, external investors, individual digital entrepreneurs as well as the public as a whole. So far, the major issue is related to the phenomenon of adopting a particular DBM and developing the relevant business sectors with policy support.

Despite the rapid development of digital businesses and market expansion in China in recent years, there have been challenges for developing businesses in this sector (Zhu et al., 2019). There are tremendous pressures from return-hungry investors who care more about immediate returns than long-term development. In addition, the monopoly of large digital companies and fierce competition force entrepreneurs to focus on short-term survival rather than long-term sustainable development. Due to a lack of intellectual property protection, the initial digital start-ups lacked commitment to developing new technology in the long run because they were worried about being copied by other players and losing money (Liu et al., 2022; Zhu et al., 2019).

More recently, due to policy shifts and changing investor behaviour during the pandemic era, some changes have occurred, leading digital entrepreneurs to become more concerned about building capabilities, high-quality brands, and sustainable growth in both domestic and international markets. Compared with large enterprises, small- and medium-sized enterprises have the advantages of light assets, high flexibility, and fast transformation, in addition to their individual unique advantages in digital transformation. Digital start-ups can build differentiated competitive advantages in response to the pandemic. Greater government support during the pandemic era is needed for the development of digital start-ups. These circumstances present important lessons for both policy makers and business leaders for future digital business development by focusing on long-term sustainable development.

## Conclusion

New start-ups developed by digital entrepreneurs are a relatively recent phenomenon among many emerging economies. China presents a unique study from which other emerging economies can draw meaningful lessons with both positive and negative elements for future development. We believe that the findings presented here based on our qualitative research provide a holistic understanding of the antecedences, intentions, behaviours, and outcomes, influenced by both endogenous and exogenous factors, in the development of digital entrepreneurship. In addition, we also make complementary contributions to the recent research on the impact of COVID-19 on economies and SMEs by adding an analysis of the exogenous influences, entrepreneurial intentions, and actions during the COVID-19 period. Some of the key issues may have global implications beyond the boundary of China.

This study was based primarily on the perspectives of digital entrepreneurs; thus, future research should include a cross-sectional investigation with multiple stakeholders based on longitudinal and historical analyses. Moreover, the qualitative nature of the research does not allow us to imply generalizability. Therefore, quantitative research is required to verify the findings generated in this study and test our theoretical framework with a comparative analysis across several countries, including developed and developing countries, in terms of the growth of digital entrepreneurship. A better understanding of the relevant issues with a global perspective can then be advanced.
